# Exercise effects on polyp burden and immune markers in the *Apc^Min^*^/+^ mouse model of intestinal tumorigenesis

**DOI:** 10.3892/ijo.2014.2457

**Published:** 2014-05-22

**Authors:** JAMIE L. McCLELLAN, JENNIFER L. STEINER, STANI D. DAY, REILLY T. ENOS, MARK J. DAVIS, UDAI P. SINGH, E. ANGELA MURPHY

**Affiliations:** 1 Department of Pathology, Microbiology and Immunology, School of Medicine, University of South Carolina, Columbia, SC 29209, USA; 2 Department of Exercise Science, School of Public Health, University of South Carolina, Columbia, SC 29208, USA

**Keywords:** physical activity, exercise, immune markers, macrophages, T cells, colorectal cancer

## Abstract

Many observational epidemiologic studies suggest an association between exercise and colon cancer risk. The mechanisms contributing to a preventative effect of exercise on colon cancer are complex and multifaceted. Altered immune system function is one possible mechanism that has been largely unexplored. Therefore, the purpose of this study was to examine the effects of exercise on markers associated with macrophages and select T cell populations in a mouse model of intestinal tumorigenesis and to relate this to polyp characteristics. Male *Apc^Min^*^/+^ mice were randomly assigned to either sedentary (Sed) or exercise (Ex) treatment (n=6–9/group). The exercise treatment consisted of treadmill running for 1 h/day and 6 days a week at 15 m/min from 4 until 16 weeks of age. Intestinal polyps were counted and categorized by size. Mucosal tissue was analyzed for mRNA expression of overall macrophages (F4/80), for genes associated with M1 (IL-12, IL-23 and Nos2) and M2 (CD206, IL-10, IL-4, CCL17, CCL22 and Arg-1) macrophages and the macrophage chemoattractants MCP-1, fetuin A and CXCL14. Markers for cytotoxic T cells (CTLs) and regulatory T cells were also examined by measuring mRNA expression of CD8 and Foxp3, respectively. While there was no significant difference in overall polyp number between the groups (Sed, 23.3±4.3; and Ex, 16.5±4.3), Ex did have a reduction in the number of large polyps (Sed, 6.1±1.1; and Ex, 3.0±0.6) (P<0.05). This was consistent with a decrease in spleen weight (P<0.05). Similarly, Ex reduced mRNA expression of overall macrophages (F4/80) as well as markers associated with both M1 (IL-12) and M2 (CD206, CCL22 and Arg-1) subtypes (P<0.05) but there was no significant decrease in macrophage chemoattractants. CD8 expression was increased while Foxp3 expression was decreased with Ex (P<0.05). Overall the data provide important new information on immune regulation as a possible mechanism for the documented benefits of exercise training on reducing colon cancer progression.

## Introduction

Colon cancer remains a significant global health concern; it is the third most common malignancy and the fourth most common cause of cancer mortality (1–4). Annually, it accounts for approximately 600,000 deaths worldwide. However, the vast majority (~80%) of these cases can be ascribed to environmental causes and are therefore potentially preventable (5). For instance, physical inactivity has been reported to account for 10% of all colon cancer cases (6), whereas physical activity has been associated with reduced risk for incidence of colon cancer. This inverse relationship between physical activity and colon cancer risk is supported by epidemiological studies as well as controlled experimental studies using rodent models. For example, 9 weeks of treadmill running has been reported to decrease the total number of intestinal polyps as well as the number of large polyps in the *Apc^Min^*^/+^ mouse model of intestinal tumorigenesis (7). Similarly a recent study in a multiethnic colon cancer screening population reported that exercising for 1 h per week was associated with a lower prevalence of polyps and adenomas when compared to those who exercised less or not at all (8).

The mechanisms responsible for a preventative effect of exercise on colon cancer risk are complex and multifaceted. An exercise-induced alteration in immune system function is one possible mechanism that has not been widely explored. Macrophages, cells of the innate immune system, have recently emerged as major components in the development of colon cancer given their ability to produce a wide array of inflammatory mediators with pro-tumoral functions (9,10). In general, these cells have been associated with poor prognosis in colon cancer (9–14). For example, one study reported a reduction in macrophage infiltration that was consistent with a decrease in the size of colon polyps in an MCP-1 receptor deficient mouse model of colitis-associated colon cancer (15). Further, in a clinical study it was documented that macrophage accumulation within the tumor was positively correlated with stage of progression (14). Consistent with these reports, we have documented that MCP-1 deficient *Apc^Min^*^/+^ mice have decreased macrophage number in both the polyps and surrounding intestinal tissue and this was associated with a reduction in total polyp and large polyp number. Exercise has been reported to influence macrophage behavior in various disease models. For instance, it has been shown that exercise can reduce macrophage-mediated inflammation in an obese mouse model (16). Similarly, aerobic exercise reverses the arterial inflammation and macrophage infiltration that is associated with aging (17). However, while it is well established that macrophages play a significant role in the pathogenesis of colon cancer and that exercise can influence the macrophage response, there is very little evidence on the benefits of exercise on macrophage behavior in the presence of colon cancer.

T cells also play a significant role in tumorigenesis and have been shown to be altered by exercise (18,19). It is well recognized that cytotoxic T lymphocytes (CTLs) constitute one of the most important effector mechanisms of antitumor immunity (20,21). For example, adoptive transfer of CD8^+^ cells controls the growth of B16 melanoma in mice (22). On the other hand, regulatory T cells (Tregs) have been associated with accelerated tumor growth and immune evasion through their inhibitory actions on CTLs and helper T cells (23,24). The T cell response to exercise has been reported to be highly variable and is likely dependent on the mode, intensity and duration of exercise. However, to date there is no evidence on the effects of exercise on T cells in a mouse model of colon cancer.

The purpose of this investigation was to examine the effects of exercise on expression of markers associated with macrophages and certain T cell subsets in a mouse model of intestinal tumorigenesis and to relate this to polyp characteristics. We used the *Apc^Min^*^/+^ mouse, the most widely used genetic mouse model for cancer studies that involve the gastro-intestinal tract (25). Since the *Apc* gene is mutated in a large percentage of human colon cancer cases, this is a common model for studying factors that may influence progression of colon cancer. The exercise protocol was designed to mimic a lifestyle that encompasses daily physical activity; mice were exercised for 1 h/day, 6 days/week, for a 12-week period. We hypothesized that daily exercise would reduce the expression of markers associated with macrophages and alter the T cell expression profile in the tumor environment, and that this would be associated with a reduction in polyp burden.

## Materials and methods

### Animals

*Apc^Min^*^/+^ mice on a C57BL/6 background were originally purchased from Jackson Laboratories (Bar Harbor, ME). All experimental mice (*Apc^Min^*^/+^) were bred in the University of South Carolina’s Center for Colon Cancer Research (CCCR) Mouse Core Facility. Specifically, *Apc^Min^*^/+^ male mice were bred with female C57BL/6 mice to generate *Apc^Min^*^/+^ mice. Offspring were genotyped as heterozygotes by RT-PCR for the *Apc* gene by taking tail snips at weaning. The primer sequences were sense, 5′-TGAGAAAGACAGAAGTTA-3′; and antisense, 5′-TTCCACTTTGGCATAAGGC-3′. Male *Apc^Min^*^/+^ mice were used in this experiment and were maintained on a 12:12 h light-dark cycle in a low-stress environment (22°C, 50% humidity and low noise) and provided food (AIN-76A) and water *ad libitum*. All animal experimentation was approved by the University of South Carolina’s Institutional Animal Care and Use Committee.

### Exercise protocol

Male *Apc^Min^*^/+^ mice were randomly assigned to either the sedentary (Sed; n=9) or exercise (Ex; n=6) treatment. Mice in the Ex group ran on the treadmill at 15 m/min and at a 5% incline for 1 h per day (starting at 7 pm), 6 days per week from 4 to 16 weeks of age. Mice in the Sed group remained in their cages in the treadmill room throughout the exercise bouts but were exposed to similar handling and noise in an attempt to control for extraneous stresses, and were deprived of food and water during the exercise sessions.

### Tissue collection

Body weight was measured weekly from 4 to 16 weeks of age. Mice were sacrificed for tissue collection as previously described (26,27). Briefly, sections 1 and 4 of the small intestine and the large intestine (section 5) were fixed in 10% buffered formalin (Fisher Scientific, Pittsburg, PA) for 24 h. For intestinal section 3, mucosal scrapings were performed in TRIzol reagent (Invitrogen, Carlsbad, CA). Samples were stored at −80°C until analysis for the expression of markers associated with macrophage phenotype and certain T cell subsets. Previously reported findings from our laboratory have shown that intestinal section 3 has a greater polyp incidence than section 2 (26), and therefore we included only section 3 for these analyses. Visceral fat pads (retroperitoneal, epididymal and mesentery) were dissected and weighed for the measurement of total visceral fat as it has been reported that *Apc^Min^*^/+^ mice become cachectic with disease progression (28). Spleen was also weighed as splenomegaly has been shown to be associated with increased polyp number in this model (29). Blood was collected from the inferior vena cava using a heparinized syringe and blood parameters were examined immediately on fresh whole blood using a Vetscan blood analyzer (Abaxis, Union City, CA).

### Polyp counts

Formalin-fixed intestinal sections from all animals were rinsed in deionized water, briefly stained in 0.1% methylene blue, and counted by the same investigator who was blinded to the treatments. Polyps were counted under a dissecting microscope and were categorized according to size (>2, 1–2 and <1 mm).

### mRNA gene expression

Quantification of gene expression for total macrophages (F4/80), markers associated with macrophage phenotype IL-12, IL-23 and Nos2 (M1 macrophage phenotype), CD206, IL-10, IL-4, CCL17, CCL22 and Arg-1 (M2 macrophage phenotype), macrophage chemoattractants (MCP-1, fetuin A and CXCL14), and T cell subsets (CD8 and Foxp3) were performed as previously described (26,30). Briefly, mucosal tissue was homogenized under liquid nitrogen with a polytron, and total RNA was extracted using TRIzol reagent (Invitrogen). The extracted RNA (2.5 μl of sample) was dissolved in diethyl pyrocarbonate (DEPC)-treated water and quantified spectrophotometrically at 260 nm wavelength. RNA was reverse transcribed into cDNA and quantitative RT-PCR analysis was done per manufacturer’s instructions (Applied Biosystems, Foster City, CA) using TaqMan^®^ Gene Expression Assays. Quantification of cytokine gene expression was calculated using the ΔΔCT method (31).

### Statistical analysis

Analysis was done using commercially available software (SigmaStat, SPSS, Chicago, IL). A two-way repeated measures ANOVA was performed on body weight measurements. T-tests were performed on all other data. Statistical significance was set with an α-value of P<0.05. Data are presented as mean ± SEM.

## Results

### Body weight and visceral fat mass

**Figure 1:**
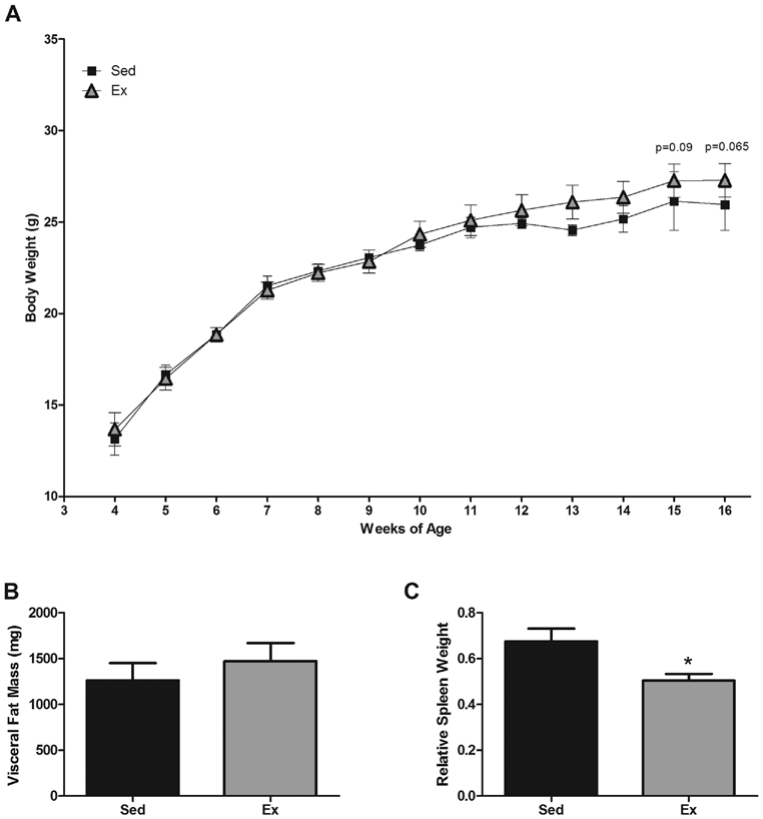
Effects of exercise on body weight, visceral fat mass and spleen weight in *Apc^Min^*^/+^ mice. Differences in (A) body weight, (B) visceral fat mass and (C) spleen weight were examined in sedentary (Sed) and exercised (Ex) *Apc^Min^*^/+^ mice (n=6–9/group) at 16 weeks of age. Values are means ± SEM. ^*^P<0.05 significantly different.

It has been reported that *Apc^Min^*^/+^ mice develop cachexia that is positively correlated with polyp burden (32). Therefore, we examined the potential benefits of exercise on body weight and visceral fat mass in the *Apc^Min^*^/+^ mouse. At 15 and 16 weeks of age, there was an apparent difference (~1–2 g) between the groups; the Sed group weighed 26.1±1.6 and 25.9±1.4 g at 15 and 16 weeks, respectively, whereas the Ex group weighed 27.3±0.9 and 27.3±0.9 g, respectively (P<0.1) (Fig. 1A). However, this did not quite reach statistical significance. Visceral fat tissue (retroperitoneal, epididymal and mesentery) was collected at sacrifice and weighed to determine any influence of exercise on cachexia-related fat mass. There was no protective effect of exercise on total visceral fat pad weight (Sed, 1262.2±189.2 mg; Ex, 1471.2±197.7 mg) (Fig. 1B).

### Spleen weight

Increased spleen weight has been positively associated with polyp burden in this model (29). Therefore, spleens were harvested during sacrifice at 16 weeks of age, weighed and expressed relative to body weight. Exercise significantly decreased spleen weight versus the Sed group (0.5±0.0 versus 0.68±0.01 mg/kg, respectively) (P=0.05) (Fig. 1C).

### Complete blood count

**Figure 2:**
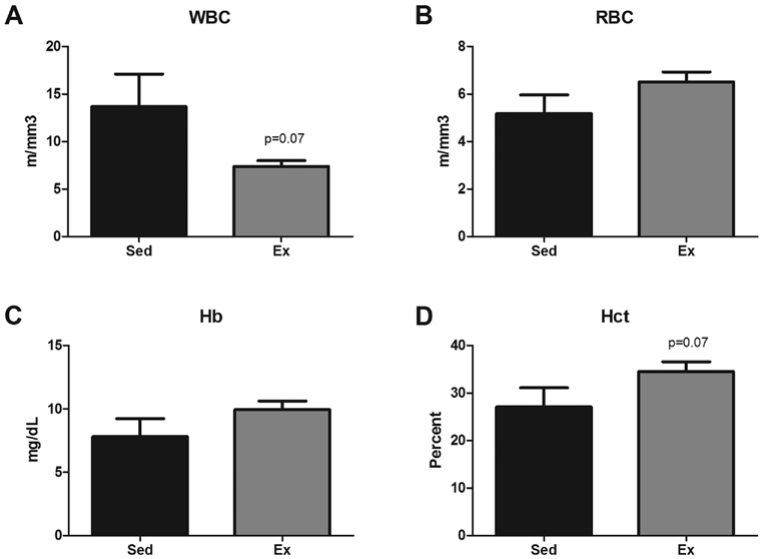
Effects of exercise on WBCs, RBCs, Hb and Hct in *Apc^Min^*^/+^ mice. Differences in (A) WBC count, (B) RBC count, (C) Hb and (D) Hct were examined in sedentary (Sed) and exercised (Ex) *Apc^Min^*^/+^ mice (n=6–9/group) at 16 weeks of age. Values are means ± SEM.

A complete blood count was performed at sacrifice as both white blood cell (WBC) and red blood cell (RBC) counts have been shown to be altered during progression of intestinal tumorigenesis in this mouse model (Fig. 2) (27). WBC count at 16 weeks of age tended to be decreased by exercise (13.7±3.2 versus 7.4±0.6 m/mm^3^) (P=0.07). Similarly, there was a trend for an exercise-induced increase in hematocrit (Hct) compared to Sed (34.6±2.0 versus 27.1±3.8%) (P=0.07). But there were no apparent differences among the groups for RBCs or hemoglobin (Hb).

### Polyp incidence

**Figure 3:**
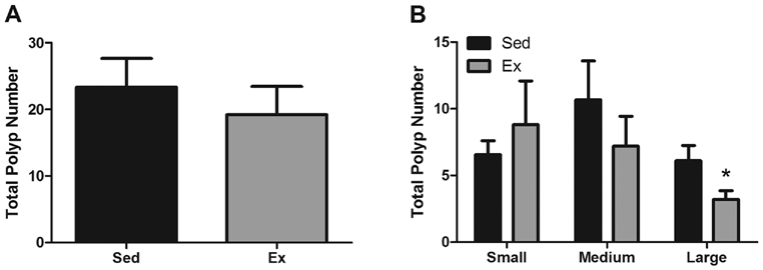
Effects of exercise on polyp number and size in *Apc^Min^*^/+^ mice. Differences in (A) total polyp number and (B) polyp size were examined in sedentary (Sed) and exercised (Ex) *Apc^Min^*^/+^ mice (n=6–9/group) at 16 weeks of age. Values are means ± SEM. ^*^P<0.05 significantly different.

At 16 weeks of age, mice (Sed and Ex) were sacrificed, intestinal tissue was harvested and polyps were counted on formalin-fixed, methylene blue-stained sections. Overall polyp number (sections 1, 4 and 5) was not significantly changed by exercise (23.3±4.3 versus 19.2±4.2 for Sed and Ex, respectively) (Fig. 3A). To examine polyp size (Fig. 3B), we counted and classified polyps as being large (>1 mm in diameter), medium (<2>1 mm in diameter) or small (<1 mm in diameter). Interestingly, we found a significant reduction in the number of large polyps with exercise; the Ex group had 48% fewer large polyps than the Sed group (3.2±0.7 versus 6.1±1.1) (P<0.05) but there were no significant differences in the number of small or medium polyps.

### Macrophage number and phenotypic markers

**Figure 4:**
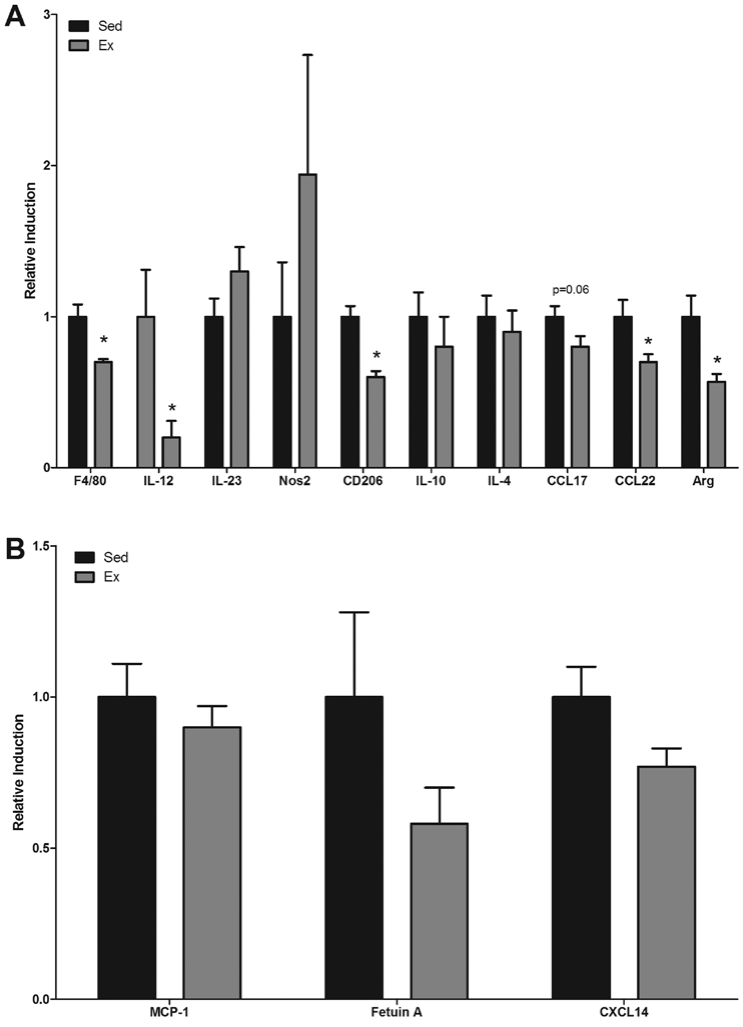
Effects of exercise on gene expression of M1 and M2 associated phenotypic macrophage markers in *Apc^Min^*^/+^ mice. Differences in gene expression of (A) F4/80, M1 macrophage markers (IL-12, IL-23, and NOS2), M2 macrophage markers (CD206, IL-10, IL-4, CCL17, CCL22 and Arg) and (B) macrophage chemattractants (MCP-1, Fetuin A, and CXCL14) were examined in sedentary (Sed) and exercised (Ex) *Apc^Min^*^/+^ mice (n=6–9/group) at 16 weeks of age in mucosal scrapings. Values are means ± SEM. ^*^P<0.05 significant difference.

Gene expression of macrophage phenotypic markers IL-12, IL-23 and Nos2 (M1 macrophage phenotype), CD206, IL-10, IL-4, CCL17, CCL22 and Arg-1 (M2 macrophage phenotype), were examined in the mucosal scrapings (Fig. 4A) of intestinal tissue. Data were normalized to fold-change from Sed mice. There was a significant decrease in mRNA expression of F4/80, a general macrophage marker (P<0.05). Similarly, there was a decrease in mRNA expression of the M2 associated macrophage markers, CD206, CCL22 and Arg in the Ex mice (P<0.05), and a trend for a decrease in CCL17 (P<0.06). Even though not all markers associated with M2 macrophages were significantly reduced with exercise, all were consistently decreased. IL-12, a marker associated with the M1 macrophage phenotype was also decreased in the Ex mice (P<0.05) but there was no change in IL-23 or Nos2. Gene expression of macrophage-associated chemokines MCP-1, fetuin A and CXCL14 were also examined in the mucosal scrapings. While mRNA expression of each of these markers was decreased with exercise (Fig. 4B), statistical significance was not reached.

### Changes in T cell expression

Given the role of T cell subsets in tumorigenesis, we also performed gene expression analysis of markers associated with CTLs (CD8) and Tregs (Foxp3) (Fi. 5). CD8, a marker for CTLs that represents one of the most important effector mechanisms of antitumor immunity, was increased with exercise (P<0.05). Conversely, Foxp3, a marker for Tregs that are known to suppress immune function and that have been associated with increased tumorigenesis, was decreased with exercise (P<0.05).

## Discussion

There is an inverse relationship between physical activity and colon cancer risk (33). A multitude of mechanisms, including immune function dysregulation, have been implicated in this response. Macrophages and T cells play a significant role in the pathogenesis of colon cancer and exercise can influence the actions of these cells; however, there is very little evidence on the benefits of exercise on macrophage and T cell responses in the settings of colon cancer. We examined the effects of exercise on markers associated with macrophages and select T cell subsets in a mouse model of intestinal tumorigenesis in relation to polyp characteristics. Overall, certain markers associated with both the M1 and the M2 macrophage phenotype were reduced in *Apc^Min^*^/+^ mice following exercise. Additionally, exercise resulted in an increased expression of CD8 and decreased expression of Foxp3, markers for CTLs and Tregs, respectively. These alterations in immune cell parameters following exercise training were accompanied by a decrease in the percentage of large polyps.

Animal models provide a tool to examine the effects of exercise on colon cancer in an experimental environment in which the type and intensity of exercise can be controlled. They allow for detailed study of stage-specific responses to exercise, and help to identify the optimal mode, intensity and duration of exercise. The benefits of exercise on colon cancer risk have been well documented in the *Apc^Min^*^/+^ mouse model of intestinal tumorigenesis (7,28,34,35). For example, 9 weeks of treadmill running has been reported to decrease the total number of intestinal polyps by 29% as well as the number of large polyps (38%) in male mice in this model (36). Similarly, exercise was reported to reduce total intestinal polyp number by 50% and the number of large polyps by 67% in this same model (34). Our findings are somewhat consistent with these investigations in that we report a 48% reduction in the number of large polyps. In contrast to the findings by Baltgalvis *et al* (7), we did not find a significant reduction in the number of total polyps; however, this is likely due to the smaller sample size in our study and/or to the slightly lower intensity of the exercise protocol (15 versus 18 m/min). Nonetheless, the benefits of regular exercise training in the *Apc^Min^*^/+^ mouse model of intestinal tumorigenesis are evident across studies and it is clear from our findings and those of others that exercise plays a larger role in reducing the progression of growth as opposed to the initiation of development, at least in this model.

In addition to polyp characteristics, we measured body weight, fat mass, spleen weight and markers of anemia. These outcomes have been associated with increased tumorigenesis and ultimately poorer prognosis in the *Apc^Min^*^/+^ mouse (27,32). Previous published data have shown that *Apc^Min^*^/+^ mice develop cachexia that is positively correlated with polyp burden (32). In our study, the exercise mice tended to be heavier than the sedentary mice at 15 and 16 weeks of age, although this did not reach statistical significance. Further, there were no differences in fat mass between the groups. However, the lack of positive findings is likely due to the timing of sacrifice as mice were sacrificed prior to the onset of severe cachexia. This was done to eliminate any possible influence of cachexia or illness on the ability to perform the exercise protocol. Therefore, it is not surprising that we did not see a statistically significant effect of exercise at these time points. Spleen weight has been associated with increased polyp number and systemic inflammation (36). Our data indicate a reduction in spleen weight with exercise. This is consistent with previously reported literature; Baltgalvis *et al* (36) also reported a reduction in spleen weight in male *Apc^Min^*^/+^ mice following exercise. We have recently reported an increase in markers of anemia in this mouse model (27). While exercise did prevent the characteristic decrease in Hct in these mice, the effect was not found to be statistically significant. Again, this is likely due to the timing of sacrifice as in our previous study mice were sacrificed at 18 weeks, a time in which the disease is much more severe. Thus, any benefits of exercise would likely be more evident had the mice been sacrificed at a later time point.

We next examined the effects of exercise on markers associated with macrophages in the mucosal tissue. Macrophages can represent up to 50% of the tumor mass producing a wide array of inflammatory mediators with pro-tumoral functions (9,10). Further, abundance of tumor associated macrophages has been associated with poor prognosis in colon cancer (9–14). Our data show a reduction in the expression of F4/80, an overall macrophage marker, with exercise. This is consistent with a previous study by Baltgalvis *et al* that reported a reduction in macrophage number following exercise training in this model (7). Because it is now well accepted that macrophages constitute an extremely heterogeneous population that is divided into two main classes (M1 and M2) (10), we next examined the effects of exercise on expression of markers associated with both the M1 and M2 phenotype. In general, it is thought that M1 macrophages are cytotoxic against neoplastic cells, whereas M2 macrophages exert pro-tumoral functions (10). We report the novel finding that exercise reduces the expression of certain markers that are associated with the M1 (IL-12) and M2 (CD206, CCL17, CCL22 and Arg-1) phenotype in the mucosal tissue. It is important to note that given the limited available tissue, macrophage markers were not measured in the polyps themselves. However, previous data from our group show a similar response for these outcomes when comparing the mucosal tissue and polyp tissue in this model (27). While our data suggest that a reduction in both M1 and M2 macrophages with exercise is associated with a reduction in polyp growth in this model, a greater understanding of the roles of each macrophage subset within the tumor microenvironment is necessary.

Given the reduction in macrophage markers with exercise, we next examined the effects of exercise on macrophage chemoattractants. MCP-1 is a major player in macrophage chemotaxis in the *Apc^Min^*^/+^ mouse (27). In fact, we recently reported a link between macrophages and MCP-1 in this mouse model (27). Although our data indicate an exercise-induced decrease in the expression of macrophage associated markers in the mucosal tissue, we did not find a significant decrease in MCP-1. Therefore, we also examined fetuin A and CXCL14, both of which have been implicated in macrophage recruitment. Fetuin A is a recently characterized macrophage chemoattractant that is also known to play a role in macrophage polarization (37). While our results show a decrease in the expression of fetuin A that is consistent with our macrophage findings, this did not reach statistical significance. Similarly, there was no effect of exercise on CXCL14, a chemoattractant for activated tissue macrophages. It is important to point out that only a subset of macrophage chemoattractants were measured in this study, thus it is possible that exercise may have impacted other chemokines.

One of the most important effector mechanisms of antitumor immunity is the activities of CTLs (20,21). For example, growth of B16 melanoma cells can be controlled in mice following the transfer of CD8^+^ cells (22). On the other hand, Tregs have been linked to accelerated tumor growth and immune evasion due to their inhibitory actions on CTLs and helper T cells (23,24). The findings of the current study show an increased expression of CD8, and conversely, a decreased expression of Foxp3 in the mucosal tissue following exercise training. While this is the first report of a favorable effect of exercise on CTLs and Tregs in a model of colon cancer, the evidence supporting exercise-induced alterations in immune function is far reaching. In general, regular moderate exercise is thought to enhance immune function; however, these effects are likely dependent on a multitude of factors including individual characteristics, the type, intensity and duration of exercise, and the stage of cancer. Although these findings support a positive effect of exercise on the T cell profile in a cancer model, it is important to point out that whether this is due to a direct effect of the exercise on these cell populations or an indirect effect that results from the reduction in large polyp number resulting from an entirely different mechanism could not be determined from this study.

Consistent with previous reports, we show a benefit of exercise training on reducing large polyp number in the *Apc^Min^*^/+^ mouse model of intestinal tumorigenesis. This was associated with alterations in the expression of immune markers in the mucosal tissue including a reduction in markers associated with M1 and M2 macrophages, an increase CD8 and a decrease in Foxp3. Overall this data provide important new information on immune regulation as a possible mechanism for the benefits of exercise training on reducing colon cancer progression.

## Figures and Tables

**Figure 5:**
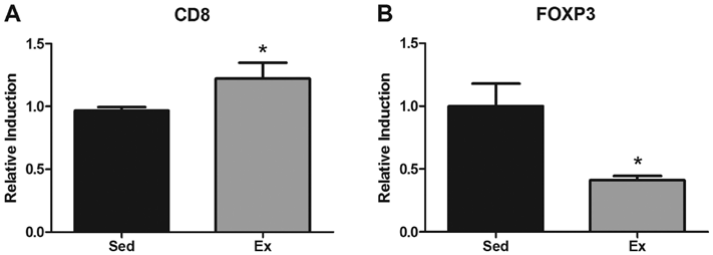
Effects of exercise on gene expression of CTL and Treg cell markers in *Apc^Min^*^/+^ mice. Differences in gene expression of (A) CD8, a marker for CTLs, and (B) Foxp3, a marker for Tregs were examined in sedentary (Sed) and exercised (Ex) *Apc^Min^*^/+^ mice (n=6–9/group) at 16 weeks of age in mucosal scrapings. Values are means ± SEM. ^*^P<0.05 significant difference.
